# Assimilation of alternative sulfur sources in fungi

**DOI:** 10.1007/s11274-018-2435-6

**Published:** 2018-03-17

**Authors:** Tomas Linder

**Affiliations:** Department of Molecular Sciences, Swedish University of Agricultural Sciences, Box 7015, 750 07 Uppsala, Sweden

**Keywords:** Desulfurization, Enzyme, Fungi, Metabolism, Sulfur

## Abstract

Fungi are well known for their metabolic versatility, whether it is the degradation of complex organic substrates or the biosynthesis of intricate secondary metabolites. The vast majority of studies concerning fungal metabolic pathways for sulfur assimilation have focused on conventional sources of sulfur such as inorganic sulfur ions and sulfur-containing biomolecules. Less is known about the metabolic pathways involved in the assimilation of so-called “alternative” sulfur sources such as sulfides, sulfoxides, sulfones, sulfonates, sulfate esters and sulfamates. This review summarizes our current knowledge regarding the structural diversity of sulfur compounds assimilated by fungi as well as the biochemistry and genetics of metabolic pathways involved in this process. Shared sequence homology between bacterial and fungal sulfur assimilation genes have lead to the identification of several candidate genes in fungi while other enzyme activities and pathways so far appear to be specific to the fungal kingdom. Increased knowledge of how fungi catabolize this group of compounds will ultimately contribute to a more complete understanding of sulfur cycling in nature as well as the environmental fate of sulfur-containing xenobiotics.

## Introduction

The element sulfur is essential for life and is found in several biomolecules including the amino acids methionine and cysteine, the redox regulator glutathione, the methylation donor *S*-adenosyl methionine and the cofactors biotin, coenzyme A, lipoic acid and thiamine. All organisms must therefore acquire sulfur from their environment for proper cellular function.

In marine environments, sulfate (SO_4_^2−^) is the predominant source of sulfur, which originates from chemical weathering of continental rock and subsequent dissolution of mineralized sulfur (Schäfer et al. 2010). Sulfur is returned to terrestrial environments through biogenic emissions of dimethylsulfide that become photooxidized into methanesulfonate in the atmosphere and deposited through precipitation (Schäfer et al. 2010; Carrión et al. 2015). Consequently inorganic species of sulfur such as sulfate are less prevalent in terrestrial soils where the main forms of sulfur consists of high-molecular weight organosulfur compounds such as sulfides, sulfoxides, sulfones, sulfonates and sulfate esters (Autry and Fitzgerald 1990; Prietzel et al. 2007). Sulfonates are also an abundant form of sulfur in marine sediments (Vairavamurthy et al. 1994). Other significant natural sources of sulfur in the environment include polycyclic organosulfur compounds found in petroleum deposits. In addition, significant quantities of anthropogenic sulfur compounds are released into the environment in the form of detergents, dyes, pesticides, pharmaceuticals and solvents.

The vast majority of previous research on sulfur assimilation in fungi has focused on the sulfur-containing amino acids methionine and cysteine as well as inorganic sources of sulfur such as sulfate, sulfite and sulfide (Marzluf 1997). However, as noted above, the predominant forms of sulfur in many natural environments populated by fungi often belong to other structural categories of compounds, which will be collectively referred to as “alternative” sulfur sources throughout this review. The term alternative sulfur source is defined here as a structurally diverse group of sulfur-containing compounds that could potentially serve as sources of sulfur but are themselves not metabolic intermediates of the central sulfur assimilation pathway in fungi. Alternative sulfur sources therefore require desulfurization, which entails the enzymatic separation of the sulfur group (predominantly in the form of the oxyanions sulfite or sulfate) from the remaining molecular backbone before the constituent sulfur can be assimilated. The vast majority of alternative sulfur sources considered in this review are organosulfur compounds. However, there are also described alternative sulfur sources that lack carbon altogether such as sulfamic acid (Linder 2012).

This review will mainly focus on our current understanding of fungal desulfurization processes from the perspective of sulfur assimilation. However, there are other conditions under which desulfurization reactions may occur. Enzymes produced by lignolytic fungi have been shown to oxidize the sulfur residues within heterocyclic organosulfide compounds (Bezalel et al. 1996; Schreiner et al. 1988; Aranda et al. 2009), which is a pre-requisite for their desulfurization. Whether these enzymes also play a direct role in sulfur assimilation for lignolytic fungi remains to be determined as these studies were carried out either using purified enzymes or in the presence of a preferred sulfur source such as sulfate or sulfur-containing amino acids. Some fungi have been reported to oxidize the sulfur residues within organosulfur compounds as part of a xenobiotic response (Ichinose et al. 2002). It is possible that some desulfurization pathways play a dedicated role in the detoxification of sulfur-containing plant protection compounds such as allicin and glucosinolate-derived isothiocyanate.

Both of the scenarios described above involve broad-specificity oxygenases such as cytochrome P450 monooxygenases (CYPs). Some fungi utilize more specialized desulfurization enzymes for the assimilation of organosulfur compounds as a source of carbon rather than sulfur. For example, a strain of the filamentous ascomycete *Fusarium proliferatum* has been shown to produce a sulfatase for the assimilation of the sulfated algal polysaccharide fucoidan as a source of carbon (Shvetsova et al. 2015). A related scenario, which entails fungal desulfurization without sulfur assimilation by the fungus itself, is the mobilization of alternative sulfur sources by fungal symbionts for the benefit of their partner organisms such as land plants (Gahan and Schmalenberger 2014). Land plants appear to lack many of the currently known enzymatic activities that are required for desulfurization.

Metabolic pathways for the assimilation of alternative sulfur sources in bacteria are fairly well understood while corresponding studies among fungi remain scarce. However, many of the bacterial enzymes that play a role in the assimilation of alternative sulfur sources have fungal homologs (Linder 2012). The present review has been organized into three main sections according to the three corresponding categories of sulfur compounds that reflect the chemical environment of the sulfur group. The first such category consists of sulfides, sulfoxides, sulfones and sulfonates, which all contain C–S bonds (R–C–S–R′). The second category consists of sulfate esters, which contain a carbon and sulfur atom separated by an oxygen atom (R–C–O–S–R′). The third category consists of sulfonamides and sulfamates, which contain N–S bonds (R–N–S–R′). There are additional structural classes of sulfur compounds that could potentially serve as sources of sulfur for fungi but the current lack of data on their assimilation does not qualify them for inclusion in the present review.

**Fig. 1:**
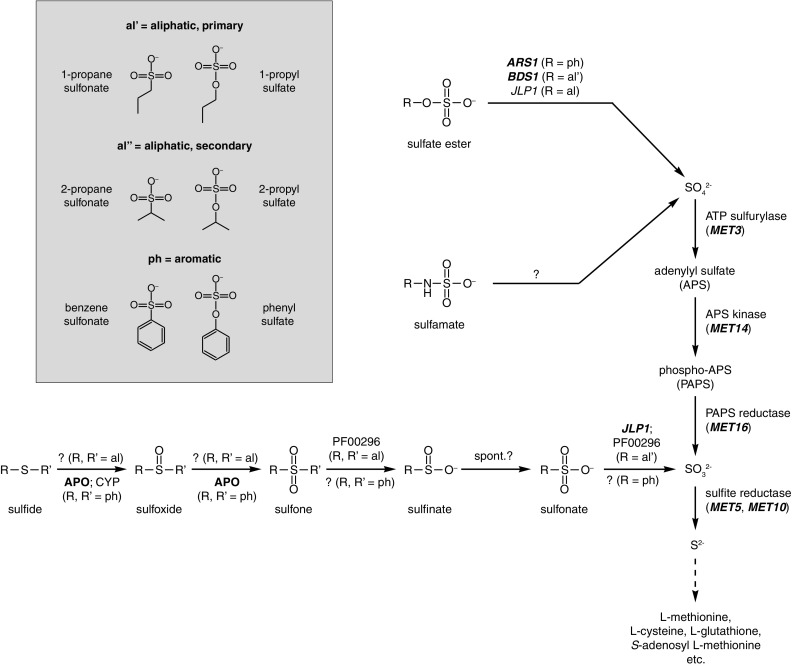
Overview of proposed pathways for desulfurization and assimilation of alternative sulfur sources in fungi. Enzymes with experimentally verified functions within the given pathways are indicated in bold font using the corresponding gene name using the standard gene nomenclature for yeast. Enzymes with predicted functions within the given pathways (for example based on sequence homology to bacterial enzymes) are indicated using regular font. The Pfam accession number PF00296 is used as a shorthand for FMNH_2_-dependent monooxygenases

The structural context of carbon side-chains adds an additional layer of complexity to the classification of sulfur compounds. This review will consider three main categories of side-chain: primary aliphatic, secondary aliphatic and aromatic (see box in Fig. 1). In the case of sulfides, sulfoxides and sulfones, the sulfur atom can be bonded to two different categories of side chains, which may require two separate enzymatic activities in order to achieve complete desulfurization.

## Sulfides, sulfoxides, sulfones and sulfonates

This category of organosulfur compounds contains sulfur atoms that share single bonds with either one (sulfonate) or two (sulfides, sulfoxides and sulfones) carbon atoms. For convenience, this group of compounds will collectively be referred to as C–S compounds in this review. Fungal desulfurization of primary aliphatic as well as aromatic C–S compounds are well established (Faison et al. 1991; Hogan et al. 1999; Baldi et al. 2003; Murakami-Nitta et al. 2003; Sood and Lal 2009; Elmi et al. 2015) while an exhaustive search of the scientific literature failed to return any published reports on the assimilation of secondary aliphatic C–S compounds by fungi. Desulfurization of both aromatic and primary aliphatic C–S compounds in fungi appears to resemble the same process in bacteria. The sulfur atom is sequentially oxidized from sulfide to sulfonate through sulfoxide and sulfone intermediates (Fig. 1; Faison et al. 1991; Murakami-Nitta et al. 2003; Sood and Lal 2009). In bacteria, the sulfone-to-sulfonate reaction involves a sulfinate intermediate that then oxidizes non-enzymatically into a sulfonate (Oldfield et al. 1997; Wicht 2016) but this process has yet to be demonstrated in fungi. Some sulfinates are desulfurized directly by bacteria without a requirement for further oxidation into sulfonates (Oldfield et al. 1997), which may also be the case in fungi. Genetic and biochemical studies in yeast suggest that assimilation of sulfonates proceed through a sulfite intermediate similarly to what happens in bacteria (Fig. 1; Uria-Nickelsen et al. 1993; Hogan et al. 1999).

At present, the only experimentally verified enzyme able to desulfurize aliphatic sulfonates is the α-ketoglutarate/Fe(II)-dependent dioxygenase Jlp1 (EC 1.14.11.17) in the baker’s yeast *Saccharomyces cerevisiae* (Hogan et al. 1999), which is homologous to the taurine dioxygenase TauD in *Escherichia coli* (van der Ploeg et al. 1996). α-Ketoglutarate/Fe(II)-dependent sulfonate dioxygenases use one oxygen atom from molecular oxygen to hydroxylate the carbon atom of the sulfonate C–S bond, which results in an unstable intermediate that then spontaneously decomposes to sulfite and the corresponding aliphatic aldehyde. The α-ketoglutarate co-substrate becomes oxidatively decarboxylated into succinate with the second oxygen atom from molecular oxygen incorporated into CO_2_ (Hogan et al. 1999). The *JLP1* gene in *S. cerevisiae* is upregulated under sulfur limitation (Boer et al. 2003). Deletion of the *JLP1* gene in *S. cerevisiae* decreased growth on sulfonates but did not abolish it altogether (Hogan et al. 1999), which may indicate the presence of other enzymes capable of desulfurizing sulfonates as *JLP1* is the only gene present in the *S. cerevisiae* genome that encodes an α-ketoglutarate/Fe(II)-dependent dioxygenase.

Flavin mononucleotide (FMNH_2_)-dependent monooxygenases (EC 1.14.14.-) are used by some bacteria for catabolism of primary aliphatic sulfones (Wicht 2016) and sulfonates (Eichhorn et al. 1999; Kertesz et al. 1999) as well as the heterocyclic organosulfide dibenzothiophene (DBT; Piddington et al. 1995). This enzyme family is conserved in fungi (Linder 2012) but has yet to be characterized by genetic or biochemical means. However, the promoter sequences of yeast genes encoding putative FMNH_2_-dependent monooxygenases contain overrepresented motifs linked to sulfur metabolism (Linder 2012), which would suggest a role in sulfur assimilation. Bacterial FMNH_2_-dependent monooxygenases are associated with NAD(P)H-dependent FMN reductases (EC 1.5.1.-; Eichhorn et al. 1999; Kertesz et al. 1999), which are also conserved in fungi (Sollner et al. 2007) but a role in sulfur assimilation has yet to be demonstrated.

**Fig. 2:**
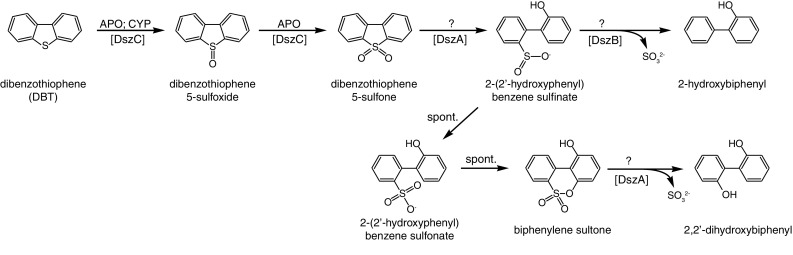
Proposed pathway for the desulfurization of DBT in fungi. Fungal enzyme families known or believed to catalyze particular reactions are indicated (Schlenk et al. 1994; Aranda et al. 2009). Names of the corresponding enzymes in *Rhodococcus* (Oldfield et al. 1997) are given in square brackets

Several studies on fungal desulfurization of aromatic C–S compounds (where the carbon atom of at least one C–S bond is located within a benzene ring) have focused on DBT due to its abundance in heavy petroleum fractions and will therefore be discussed in greater depth. The utilization of DBT as a sulfur source has previously been reported in the basidiomycete yeasts *Rhodosporidium toruloides* (Baldi et al. 2003) and *Trichosporon* (Zahra et al. 2006) as well as the filamentous ascomycetes *Exophiala spinifera* (Elmi et al. 2015) and *Stachybotrys bisbyi* (Gherbawy et al. 2016). In addition, several other fungi have been demonstrated to oxidize the constituent sulfur atom and in some cases completely desulfurize DBT through sulfoxide and sulfone intermediates analogous to aliphatic C–S compounds (Fig. 2; Faison et al. 1991; Schlenk et al. 1994; Sood and Lal 2009). As pointed in the beginning of this review, fungal DBT *S*-oxidation and desulfurization can occur indirectly during lignin degradation or as part of a xenobiotic response rather than as part of sulfur assimilation. Enzyme families shown to mediate DBT *S*-oxidation include extracellular aromatic peroxygenases (APO; EC 1.11.2.1; Aranda et al. 2009) and CYPs (Schlenk et al. 1994; Ichinose et al. 2002).

Once DBT 5-sulfone has been formed, actual desulfurization can occur. Although the exact mechanism of desulfurization has not been elucidated in fungi, it is thought to resemble that of strains of the bacterial genus *Rhodococcus*, where two separate mechanisms have been described for the desulfurization step (Oldfield et al. 1997). Following the breaking of the first C–S bond of DBT 5-sulfone by the FMNH_2_-dependent monooxygenase DszA (EC 1.14.14.22), the resulting product is thought to be 2-(2′-hydroxyphenyl)benzene sulfinate (Fig. 2). The *Rhodococcus* desulfinase DszB (EC 3.13.1.3) is then able to break the second C–S bond to produce sulfite and 2-hydroxybiphenyl (Oldfield et al. 1997). The DszB protein lacks fungal homologs (Linder 2016) but an analogous process appears to occur in some fungi since formation of 2-hydroxybiphenyl from DBT has been reported for *Candida digboiensis* (Sood and Lal 2009), *E. spinifera* (Elmi et al. 2015) and *S. bisbyi* (Gherbawy et al. 2016). A second proposed branch of DBT desulfurization pathway in *Rhodococcus* involves non-enzymatic oxidation of 2-(2′-hydroxyphenyl)benzene sulfinate to the corresponding sulfonate (Oldfield et al. 1997). Spontaneous condensation of 2-(2′-hydroxyphenyl)benzene sulfonate to biphenylene sultone subsequently allows for desulfurization by DszA to produce sufite and 2,2′-dihydroxybiphenyl. As DszA is an FMNH_2_-dependent monooxygenase, it does have fungal homologs (Linder 2012). However, it remains to be established whether this enzyme family plays a role in fungal DBT desulfurization. It is notable that the formation of 2,2′-dihydroxybiphenyl rather than 2-hydroxybiphenyl from the desulfurization of DBT has been reported in the filamentous ascomycete *Paecilomyces* (Faison et al. 1991) and the basidiomycete yeast *R. toruloides* (Baldi et al. 2003).

Studies of the catabolism of other aromatic C–S compounds remain scarce. *Paecilomyces* was shown to oxidize diphenyl sulfide into the corresponding sulfone (Faison et al. 1991). The utilization of aromatic sulfonates as sulfur sources has so far only been reported in the basidiomycete yeast *R. toruloides* (Baldi et al. 2003) and the ascomycete yeast *Lipomyces starkeyi* (Linder 2016).

## Sulfate esters

**Fig. 3:**
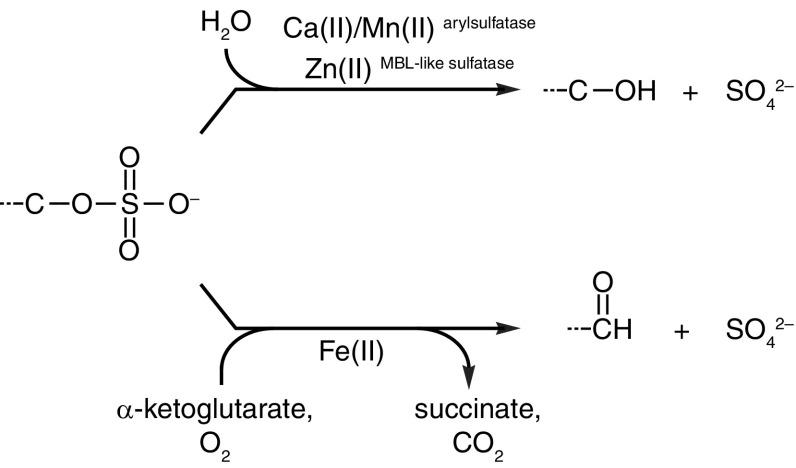
Overview of reaction mechanisms for desulfurization of sulfate esters in fungi. Metal ion requirements for each enzyme family are indicated (Hogan et al. 1999; Korban et al. 2017; Waddell et al. 2017)

There are three separate enzyme families associated with fungal desulfurization of sulfate esters, two of which have been verified by biochemical experiments. The first such enzyme family are the arylsulfatases (EC 3.1.6.1), which desulfurize aromatic sulfate esters through hydrolysis to produce sulfate and the corresponding aromatic alcohol (Fig. 3; upper reaction). This enzyme family also desulfurizes non-aromatic sulfate esters such as sulfated carbohydrates (Shvetsova et al. 2015) and choline *o*-sulfate (Østerås et al. 1998). Fungal arylsulfatases have been biochemically characterized in the filamentous ascomycetes *Aspergillus oryzae* (Sampson et al. 1975) and *F. proliferatum* (Korban et al. 2017) as well as in the ascomycete yeast *Kluyveromyces lactis* (Stressler et al. 2016). Although similar to bacterial and metazoan arylsulfatases, the fungal enzymes appear lack the characteristic Cα-formylglycine post-translational modification found in both the bacterial and metazoan enzymes (Schmidt et al. 1995; Miech et al. 1998; Korban et al. 2017).

The metallo-β-lactamase (MBL)-like sulfatase enzyme family (EC 3.1.6.-) has been demonstrated to be involved in fungal desulfurization of aliphatic sulfate esters to produce sulfate and the corresponding aliphatic alcohol (Fig. 3; upper reaction). The *S. cerevisiae* Bds1 protein is currently the only fungal enzyme belonging to this family to have been characterized through genetic and biochemical means (Hall et al. 2005; Waddell et al. 2017). Bds1 specifically desulfurizes unbranched primary aliphatic sulfate esters, which is thought to occur by hydrolysis of the C–O bond through a nucleophilic attack by an exchangeable water molecule on the carbon atom with subsequent incorporation of the water-derived oxygen atom into the resulting alcohol (Waddell et al. 2017). The *BDS1* gene appears to have been acquired by a fairly recent ancestor of *S. cerevisiae* through horizontal gene transfer from proteobacteria (Hall et al. 2005). However, the same enzyme family occurs sporadically throughout the fungal kingdom, which could indicate multiple gene transfer events from bacteria (author’s unpublished observation). The *BDS1* gene in *S. cerevisiae* is upregulated under sulfur limitation (Boer et al. 2003).

The third enzyme family implicated in the desulfurization of sulfate esters are α-ketoglutarate/Fe(II)-dependent dioxygenases mentioned previously. Alkylsulfatase activity has previously been reported for the bacterial α-ketoglutarate/Fe(II)-dependent dioxygenase AtsK (Kahnert and Kertesz 2000). The catalytic mechanism of the bacterial AtsK enzyme is thought to be analogous to that of sulfonate-specific dioxygenases. The carbon atom adjacent to the sulfate group becomes hydroxylated by one oxygen atom derived from molecular oxygen to produce the corresponding 1-hydroxyalkyl sulfate, which spontaneously decomposes into sulfate and the corresponding aliphatic aldehyde (Fig. 3; lower reaction). The co-substrate becomes oxidatively decarboxylated into succinate as described previously. The bacterial AtsK enzyme has been shown to utilize a number of α-ketodicarboxylic acids as co-substrates (Kahnert and Kertesz 2000), which may also be the case for some fungal α-ketoglutarate/Fe(II)-dependent dioxygenases.

At the time of writing, no direct experimental evidence have demonstrated the involvement of fungal α-ketoglutarate/Fe(II)-dependent dioxygenases in the desulfurization of sulfate esters. However, yeast sulfur assimilation data has demonstrated that several yeast species are capable of utilizing sulfate ester as sulfur sources without possessing either arylsulfatases or MBL-like sulfatases (Linder 2012). The fact that many fungal genomes contain multiple genes encoding putative α-ketoglutarate-dependent dioxygenases—often flanked by upstream promoters enriched in known sulfur regulatory sequence motifs (Linder 2012), could indicate that this enzyme family in involved in the desulfurization of a wide range of sulfur compounds.

## Sulfonamides and sulfamates

Very little is known regarding the assimilation of sulfonamides (R–N–SO_2_–R′) and sulfamates (R–N–SO_3_^−^) in fungi. The simplest N–S compound is sulfamic acid (NH_2_SO_3_H) and a number of ascomycete yeasts have been shown to utilize the ammonium salt of sulfamic acid as a sulfur source (Linder 2012). The same species of yeast are also able to utilize the sodium salt of *N*-cyclohexylsulfamic acid as a sulfur source (author’s unpublished data). Sulfamates are thought to be assimilated through a sulfate intermediate (Fig. 1) as the enzyme ATP sulfurylase (encoded by the *MET3* gene in yeast) is essential for the utilization of sulfamate as a sulfur source (Linder 2017). The enzyme responsible for desulfurization of sulfamates remains to be identified. Both human and bacterial arylsufatase-type enzymes have previously been shown to be involved in the desulfurization of sulfamate-containing polysaccharides such as heparan sulfate (Scott et al. 1995; Myette et al. 2009). However, the presence of arylsulfatase-family enzymes does not correlate with the ability to utilize sulfamate as a sulfur source among yeasts (Linder 2012, 2017). Conversely, the utilization of sulfamate-containing polysaccharides as sulfur sources has not yet been established in fungi, which does not exclude that fungal arylsulfatase-type enzymes could play a role in the desulfurization of such compounds.

## Future research directions

Although fungal desulfurization of alternative sulfur sources has been well known for decades, the field remains largely unexplored. Much of the current model of desulfurization pathways in fungi (Fig. 1) is still based on speculation informed by previous work on the corresponding pathways in bacteria. A number of research priorities can be identified to improve our current understanding of how assimilation of alternative sulfur sources functions in fungi.

The first priority is to identify the enzymes that are responsible for desulfurization of “orphan” sulfur sources such as sulfamate and aromatic sulfonates. Global expression analysis of selected fungi in the presence of such sulfur sources compared to a control sulfur substrate such as sulfate or methionine would be a straightforward approach towards identification of these enzymes. The second priority is to address the need for mechanistic studies that employ reverse genetics, in vitro enzymology and protein structure determination. This should include both enzymes with confirmed function in sulfur assimilation as well as enzymes predicted to play a role in sulfur assimilation based on their homology to previously described bacterial enzymes (e.g. putative FMNH_2_-dependent sulfonate/sulfone monooxygenases, putative α-ketoglutarate/Fe(II)-dependent sulfate ester dioxygenases). Such studies need to be systematic and include structurally comprehensive sets of substrates in order to take account of variation in side-chain structure and size (e.g. aliphatic vs. aromatic, primary vs. secondary, side-chain length). The third priority is to investigate the role of dedicated membrane-bound transporters in the ability of fungi to utilize alternative sulfur sources. A sulfonate transporter was recently identified in *S. cerevisiae* (Holt et al. 2017). The fourth priority is to continue the exploration into what further structural classes of sulfur compounds can be utilized as sulfur sources by fungi.

In conclusion, it is well established that fungi play a fundamental role in nutrient cycling thanks to their ability to degrade complex organic substrates. It is conceivable that the same metabolic versatility allows fungi to also participate in the global sulfur cycle through the mobilization of “alternative” sulfur reserves in terrestrial ecosystems. As the enzyme systems responsible for these metabolic activities in fungi are identified and characterized, it will be possible to monitor these processes in situ using sequencing-based methods. This will in turn also enable studying the role of fungi in catabolizing sulfur-containing xenobiotics in the natural environment. The research field of fungal catabolism and assimilation of alternative sulfur sources is still in its infancy with many key discoveries yet to be made.

## Abbreviations

APO: Aromatic peroxygenase
CYP: Cytochrome P450 monooxygenase
DBT: Dibenzothiophene
FMN/FMNH_2_: Flavin mononucleotide
